# A survey on the disposal of blood-contaminated tampon after dental extraction

**DOI:** 10.1186/s40064-016-3210-5

**Published:** 2016-09-07

**Authors:** Jie Dai, Yong-Ping Zhang, Wen-Min Wang, Xu-Ming Luo, Wen-Jie Zhuo, Wei-Jiang Yang, Ling-Zhi Zhang

**Affiliations:** 1 Department of Stomatology, Luqiao Branch of Taizhou Hospital of Zhejiang Province, Taizhou, 318050 China; 2 Department of Hospital Infection Control, Luqiao Branch of Taizhou Hospital of Zhejiang Province, Taizhou, 318050 China

**Keywords:** Blood-contaminated tampon, Cross infection, Dental extraction, Patients, Dentists

## Abstract

**Objective:**

This paper was to assess the risk for cross infection caused by blood-contaminated tampon after dental extraction and whether this risk was reduced after relevant education towards both dentists and patients.

**Methods:**

From December 2014 to April 2015, a survey was conducted in dentists and patients randomly before and after relevant education. The questionnaire is being revised for this survey based on learning from Chatzoudi and Franklin’ survey. This survey was approved by the institutional review board, and all participants were voluntary and all responses were anonymous.

**Results:**

Only 2.82 % of dentists provided patients with the postoperative-advices regarding how to dispose of blood-contaminated tampon at the first time and 47.10 % at the second time (*P* < 0.01). Only 1.41 % of dentists given special postoperative-advices regarding disposal of tampon to patients with blood-transmitted diseases at the first time and 24.64 % at the second time (*P* < 0.01). Before education, most patients were lack of nosocomial infection knowledge. After education, 22.4 % of patients threw the blood-contaminated tampon away in a proper way (*P* < 0.01). 66.67 % of them washed hands immediately and thoroughly after they touched the tampon (*P* < 0.05), 92.71 % knew the blood-contaminated tampon can cause cross-infection (*P* < 0.01), and 80.21 % knew how to dispose of the blood-contaminated tampon correctly (*P* < 0.01).

**Conclusion:**

The high risk of cross infection caused by blood-contaminated tampon is evident, and a series of measures is proposed to control it. There is a need to improve both dentists’ and patients’ awareness, enhance the education of doctors and perfect the policies and guidelines.

## Background

Infection control is a major issue in the dental practice and has attracted increasing attention. Many surgical dressings, such as tampon, cotton ball, and gauze, are usually used to control bleeding after dental extraction or oral surgery, and patients are usually told to bite them for half an hour and then dispose of them when the bleeding stops. However, the places and the ways to deal with blood-contaminated tampon, as well as the safety of disposal, are usually ignored finally.

To date, most government administrative organizations and many health organizations have established strict and useful infection control policies and guidelines for the proper disposal of clinical waste (Oosthuysen et al. 2014). However, all of them focus on the education of dentists, but not patients, and there are no guidelines regarding the proper disposal of the gauze which are used to control bleeding after dental extraction and other oral surgeries in patients. What is worse, most patients lack the necessary knowledge on the nosocomial infection (Lin et al. 2008; Wang et al. 2012), many patients never realize their blood as a possible source of infection (Buddeberg 1980), and, even they has realized it, many patients with blood-transmitted diseases are not reluctant to disclose their health status because of their fear of discrimination (Charbonneau et al. 1999). A study of Franklin and Laskin (2014) pointed out, although all dentists provided patients with both written and verbal postoperative-advices, but these postoperative-advices seldom indicated how to dispose of blood-contaminated tampon, and patients with blood-transmitted diseases rarely got special advices. A study of Chatzoudi (2009) found that most patients threw the gauze away in an improper way and half of patients touched the gauze with their bare hands before its final disposal. Both studies indicate that there is high risk for cross infection caused by blood-contaminated tampon after dental extraction, and most dentists and patients still have not paid enough attention to it. However, the issue that whether doctors and patients will pay enough attention and take adequate measures after they are educated is not involved in both studies.

This study was to assess the risk for cross infection caused by blood-contaminated tampon after dental extraction, and determine whether dentists had provided patients with adequate postoperative-advices to avoid the cross infection after dental extraction, whether patients had gotten and understood postoperative-advices and then practiced, and whether the risk for cross infection would be reduced after dentists and patients were educated.

## Methods

### Questionnaire design

The questionnaire is being revised for this survey based on learning from Chatzoudi and Franklin’ survey. The questionnaire for dentists includes 6 closed questions (Chatzoudi 2009): whether they provide verbal postoperative-advices to patients; whether they provide written postoperative-advices to patients; whether they give patients extra tampon in case the initial tampon does not adequately control the bleeding; whether their postoperative-advices give an explanation to their patients about how to dispose of the blood-contaminated tampon; whether they give special advices on disposal of tampon to the patients with blood-transmitted diseases, such as hepatitis, AIDS, and syphilis; whether they provide self-sealing plastic bag to patients. The questionnaire for patients includes 5 closed questions (Franklin and Laskin 2014): the most likely ways in which they finally throw the blood-contaminated tampon; whether they wash theirs hands immediately and thoroughly after they touch the tampon by hand; whether they know the blood-contaminated tampon may cause cross infection; whether the patients know how to dispose of the blood-contaminated tampon correctly; whether they dispose of the blood-contaminated tampon carefully if they have blood-transmitted disease.

### Survey administration

The survey was conducted in both dentists and patients face -to- face. A total of 300 randomly selected dentists were recruited on spot from Taizhou City (a city of Zhejiang province in China) between December 2014 and April 2015, and 142 completed this survey with questionnaire with the response rate of 47.33 %. Three months later, the same survey was conducted in these dentists, and 138 dentists responded with the response rate of 97.18 %. In addition, a survey was also conducted in 200 randomly selected patients whose dentists responded to the above survey at the first survey. They used tampon to control the postoperative bleeding and were given postoperative-advices immediately after dental extraction or other oral surgeries. They were questioned by telephone on the day after dental extraction or other oral surgeries. Finally, 187 of 200 patients participated in this survey with the response rate of 93.50 %. Three months later, the same survey was conducted in 200 randomly selected patients whose dentists responded and had taught them how to dispose of tampon at the second time. In addition, the same survey was conducted in 200 randomly selected patients whose dentists responded and had not taught them how to dispose of blood-contaminated tampon at the second time. Finally, 382 patients responded with the response rate of 95.50 %.

### Sampling procedure

The survey for dentists was conducted by using the cluster sampling method, and the survey for patients was conducted by using the simple random sampling method.

### Ethical consideration

This was a cross sectional study approved by the Ethics Committee of Luqiao Branch of Taizhou Hospital of Zhejiang Province. Informed consent was obtained from every subject.

### Statistical analysis

Statistical analyses were conducted by using the Statistical Package for Social Science, version 16.0 (SPSS Inc., Chicago, IL, USA). Chi square test was used for the comparisons between groups. A value of *P* < 0.05 was considered statistically significant.

## Results

Results showed that all the dentists provided patients with verbal postoperative-advices (*P* > 0.05) and most provided patients with extra tampon in case the initial tampon did not adequately control the bleeding in both surveys (*P* > 0.05). 98 of 142 (69.01 %) respondents provided patients with a postoperative-advices at the first time and 115 of 138 (83.33 %) at the second time (*P* < 0.01). However, only 4 of 142 (2.82 %) respondents indicated that the postoperative-advices provided to patients told patients how to dispose of blood-contaminated tampon at the first time and 65 of 138 (47.10 %) at the second time (*P* < 0.01). Moreover, only 2 of 142 (1.41 %) respondents indicated that they had given special postoperative-advices regarding the disposal of tampon to patients with blood-transmitted diseases at the first time and 34 of 138 (24.64 %) at the second time (*P* < 0.01). The proportions of dentists giving postoperative-advices regarding the disposal of tampon and giving special postoperative-advices to patients with blood-transmitted diseases slightly increased at the second time (44.28 and 23.23 %, respectively), which indicates that most dentists still do not paid enough attention to both issues. What is worse, most dentists did not provide patients with self-sealing plastic bag used for disposal of tampon in both surveys (*P* > 0.05). Although most dentists claimed that it was a good way to avoid cross infection, they did not provide self-sealing plastic bag because there was no proper and finished product on sale, and some dentists claimed that it was not recommended by infection control policies and guidelines. These results suggest that the condition is not optimistic in terms of dentists, even when they were educated (Table 1).

**Table 1:** Survey on postoperative-advices from dentists

Question	First (n = 142)	Second (n = 138)	χ^2^	*P*
Do you provide patients with verbal postoperative-advices after dental extraction or other oral surgeries?				
Yes	142 (100.00 %)	138 (100.00 %)	/	>0.05
No	0 (0.00 %)	0 (0.00 %)		
Besides the verbal postoperative-advices, do you provide patients with a postoperative-advice sheet after dental extraction or other oral surgeries?				
Yes	98 (69.01 %)	115 (83.33 %)	7.8834	<0.01
No	44 (30.99 %)	23 (16.67 %)		
Besides the postoperative-advices, do you provide patients with extra tampon to control the postoperative bleeding after surgeries?				
Yes	86 (60.56 %)	96 (69.57 %)	0.5982	>0.05
No	46 (32.39 %)	42 (30.43 %)		
Do the postoperative-advices provide information on how to dispose of blood-contaminated tampon?				
Yes	4 (2.82 %)	65 (47.10 %)	73.9092	<0.01
No	138 (97.18 %)	73 (52.90 %)		
Do you give special postoperative-advices regarding the disposal of tampon to patients with blood-transmitted diseases, such as hepatitis, AIDS and so on?				
Yes	2 (1.41 %)	34 (24.64 %)	33.7057	<0.01
No	140 (98.59 %)	104 (75.36 %)		
Do you provide patients with self-sealing plastic bag for disposal of tampon?				
Yes	142 (100.00 %)	136 (98.55 %)	0.5329	>0.05
No	0 (0.00 %)	2 (1.45 %)		

Most of patients threw blood-contaminated tampon away in an improper way, approximately half of the patients recalled having touched the tampon by hand and only approximately half of them washed their hands immediately and thoroughly after having touched the tampon by hand. The reason for touching the tampon was that they felt that it was dirty, they could not bear nausea or they didn’t follow advices carefully or forgot and so on. Most patients didn’t wash their hands because they could not find the washing room immediately, they were on public transportation or they forgot. Although more than sixty percent of patients knew the blood-contaminated tampon can cause cross infection, patients seldom knew how to dispose of the blood-contaminated tampon correctly. Moreover, about sixty percent of patients with blood-transmitted disease didn’t dispose of the blood-contaminated tampon carefully and specially. These conditions were improved at the second time after patients being educated. 22.4 % patients threw blood-contaminated tampon away into the clinical waste box or the self-sealing plastic bags (*P* < 0.01). Half of patients recalled having touched the tampon by hand and 66.67 % of them washed their hands immediately and thoroughly (*P* < 0.05). 92.71 % of patients knew the blood-contaminated tampon can cause cross infection (*P* < 0.01), and 80.21 % of patients knew how to dispose of the blood-contaminated tampon correctly (*P* < 0.01). These findings suggest that advices from dentists play an important role in improving it. After education, 79.41 % of patients with blood-transmitted disease disposed of the blood-contaminated tampon carefully and specially (*P* < 0.01), but remaining 20.59 % still did not, which was serious potential risk for cross infection. Of 9 patients who were provided with self-sealing plastic bags from their dentists, 7 threw blood-contaminated tampon away into the plastic bags, 1 said there’s not enough time to throw them away into plastic bags because of nausea and vomiting, and 1 said he did not remember. Results are shown in Table 2.

**Table 2:** Survey on how to dispose of blood-contaminated tampon in pateints

Question	First (n = 187)	Second		χ^2^	*P*
		Un-educated (n = 190)	Educated (n = 192)		
Where did you finally throw the tampon postoperatively?					
Clinical waste box	11 (5.88 %)	15 (7.89 %)	36 (18.75 %)	17.7581	<0.01
Self-sealing plastic bag	0 (0.00 %)	0 (0.00 %)	7 (3.65 %)		
Clinic’s toilet	16 (8.56 %)	20 (10.53 %)	23 (11.98 %)		
Roads’ dustbin	62 (33.16 %)	54 (28.42 %)	45 (23.44 %)		
Home’ wastebin	71 (37.97 %)	76 (40 %)	48 (40.00 %)		
Other	15 (8.02 %)	14 (7.37 %)	19 (9.90 %)		
Forget	12 (6.42 %)	11 (5.79 %)	14 (7.29 %)		
Had you touched the tampon by hand?					
Yes	98 (52.41 %)	112 (58.95 %)	96 (50 %)	2.4925	>0.05
No	79 (42.25 %)	70 (36.84 %)	84 (43.75 %)		
Forget	10 (5.35 %)	8 (4.21 %)	12 (6.25 %)		
If yes, whether you wash your hands immediately and thoroughly?					
Yes	57 (58.16 %)	54 (48.21 %)	64 (66.67 %)	6.0469	<0.05
No	32 (32.65 %)	45 (40.18 %)	25 (26.04 %)		
Forget	9 (9.18 %)	13 (11.61 %)	7 (7.29 %)		
Do you know the blood-contaminated tampon may cause cross infection?					
Yes	126 (67.38 %)	117 (61.58 %)	178 (92.71 %)	52.616	<0.01
No	61 (32.62 %)	73 (38.42 %)	14 (7.29 %)		
Do you know how to dispose of the blood-contaminated tampon correctly?					
Yes	0 (0.00 %)	3 (1.58 %)	154 (80.21 %)	243.897	<0.01
No	187 (100.00 %)	187 (98.42 %)	38 (19.79 %)		
If you are a patient with blood-transmitted disease, do you dispose of the blood-contaminated tampon correctly?					
Yes	10 (35.71 %)	12 (41.38 %)	27 (79.41 %)	9.5995	<0.01
No	18 (64.29 %)	17 (58.62 %)	7 (20.59 %)		

## Discussion

Our results indicated that, under the strict implementation of infection control policies and guidelines, dentists were not as compliant as we thought, which means policies and guidelines are not perfect enough. Dentists did not provide patients with adequate postoperative-advices to avoid the cross infection after dental extraction or other oral surgeries, even when dentists had been educated. The proportion of dentists giving postoperative-advices regarding the disposal of tampon and giving special postoperative-advices to patients with blood-transmitted diseases increased slightly after education, which means most dentists did not pay enough attention to these issues (Elkarim et al. 2004; Porter 1991). To prevent infections in the Department of Stomatology, some hygiene rules must be followed. It is necessary to enhance the education for dentists and revise the guidelines on how to disposal of the blood-contaminated tampon after oral surgeries (Barlean et al. 2012, 2013). In addition, it is also necessary for dentists to provide patients with both verbal and written postoperative-advices, because patients may not notice and remember what dentists say after oral surgeries.

Moreover, our results revealed that patients did not pay enough attention to the potential of cross infection caused by blood-contaminated tampon, even when they had been educated. Many patients never realized their blood as a possible source of infection. Meanwhile, theirs behaviors and reactions could not always be foreseen; they may touch the tampon with their bare hands naturally when they could not bear nausea or for many other reasons. However, they might not wash their hands immediately because they could not find washing room or they were on the way home or for many other reasons. This may be the reason why there were still fewer patients practicing as we thought, even though they had been educated before. What was worse, many patients might throw blood-contaminated tampon away at any time and any place without any protection, which was also quite unpredictable, unintentional and quite dangerous.

Recently, cross infections are no longer confined to the hospital environment, many research reported that cross infections outbreaks have been attributed to non-adherence to recommended infection-prevention procedures (Hefzy et al. 2016). Thus, measures should be taken to prevent from cross infection. First, all the patients need to be informed that their blood, saliva and other bodily fluids are possible sources of infection (Infection Control in Dentistry 2008), and they should treat them properly, even if they are completely healthy. In addition, patients with blood-borne diseases, such as hepatitis, AIDS, and syphilis, should be given special postoperative-advices. The advices are better in the written form in case the patients may forget after surgery. Meanwhile, dentists must regard all the patients as possible carriers of infectious diseases, even if they are completely healthy. Second, all the patients need to be informed that washing hands is important and the hands should be washed in a right way (da Cunha et al. 2014; Al-Khatib et al. 2015). Cross infection can be caused by contacting with not only the blood-contaminated tampons disposed by patients in clinic’s garbage bins, on the road, or at home, but objects that patients contacted with after they contacted with the blood-contaminated tampons with their bare hands, such as door handle, escalator,medical record note, wallet, car door, and even the stopcock. Therefore, all the areas in the clinical room should be cleaned at least twice daily. Hand sanitizer must be accessible to both patients and dentists in the clinc, as failure to adhere to hand hygiene is the major source of transmitting infectious diseases (Infection prevention and control in pediatric ambulatory settings 2007). Moreover, effective and frequent hand washing is the most important measure for patients to prevent infection. Finally, all the patients should be given a self-sealing plastic bag with warning signs (Franklin and Laskin 2014; Infection Control in Dentistry 2008), which should include postoperative-advices and other special advices, such as ‘‘Medical Wastes’’, ‘‘Risk of Infection, Avoid Touching’’, ‘‘Be sure the blood-contaminated tampon is safely disposed so no one else can touch it’’ and ‘‘Only YOU the patient should touch any blood-contaminated tampon’’. The self-sealing plastic bag may be used for disposal of blood-contaminated tampon and prevent from cross infection. Fortunately, our results indicated that most patients were given self-sealing plastic bag from their dentists and threw blood-contaminated tampon away into the self-sealing plastic bag.

Blood-contaminated tampon has been identified to be a potential hazard for the cross infection and the role of tampon as a carrier in cross infection should be explored and addressed further. There is a need to improve both dentists’ and patients’ awareness, strengthen the education of doctors, and perfect the policies and guidelines. In addition, dentists should provide specific advices about the proper disposal of the tampon, knowledge on hand hygiene and self-sealing plastic bag for all the patients to avoid cross infection. The potential cross infection will be avoided with the implementation of these measures.

## Conclusions

The high risk of cross infection caused by blood-contaminated tampon is evident, and a series of measures is proposed to control it. There is a need to improve both dentists’ and patients’ awareness, enhance the education of doctors and perfect the policies and guidelines.
